# *Ex vivo* and *in vivo* evidence that cigarette smoke-exposed T regulatory cells impair host immunity against *Mycobacterium tuberculosis*

**DOI:** 10.3389/fcimb.2023.1216492

**Published:** 2023-10-26

**Authors:** Xiyuan Bai, Deepshikha Verma, Cindy Garcia, Ariel Musheyev, Kevin Kim, Lorelenn Fornis, David E. Griffith, Li Li, Nicholas Whittel, Jacob Gadwa, Tamara Ohanjanyan, Matthew J. Eggleston, Manuel Galvan, Brian M. Freed, Diane Ordway, Edward D. Chan

**Affiliations:** ^1^ Department of Academic Affairs, National Jewish Health, Denver, CO, United States; ^2^ Department of Medicine, National Jewish Health, Denver, CO, United States; ^3^ Division of Pulmonary Sciences and Critical Care Medicine, University of Colorado Anschutz Medical Campus, Aurora, CO, United States; ^4^ Mycobacteria Research Laboratories, Department of Microbiology, Immunology and Pathology, Colorado State University, Fort Collins, CO, United States; ^5^ Complement Laboratory, Advance Diagnostics, National Jewish Health, Denver, CO, United States; ^6^ Department of Medicine, University of Colorado Anschutz Medical Campus, Aurora, CO, United States; ^7^ Department of Medicine, Rocky Mountain Regional Veterans Affairs Medical Center, Aurora, CO, United States

**Keywords:** adoptive transfer, CTLA-4, macrophage, autophagy, T regulatory

## Abstract

**Introduction:**

A strong epidemiologic link exists between cigarette smoke (CS) exposure and susceptibility to tuberculosis (TB). Macrophage and murine studies showed that CS and nicotine impair host-protective immune cells against *Mycobacterium tuberculosis (MTB)* infection. While CS and nicotine may activate T regulatory cells (Tregs), little is known about how CS may affect these immunosuppressive cells with *MTB* infection.

**Methods:**

We investigated whether CS-exposed Tregs could exacerbate *MTB* infection in co-culture with human macrophages and in recipient mice that underwent adoptive transfer of Tregs from donor CS-exposed mice.

**Results:**

We found that exposure of primary human Tregs to CS extract impaired the ability of unexposed human macrophages to control an *MTB* infection by inhibiting phagosome-lysosome fusion and autophagosome formation. Neutralizing CTLA-4 on the CS extract-exposed Tregs abrogated the impaired control of *MTB* infection in the macrophage and Treg co-cultures. In Foxp3^+^GFP^+^DTR^+^ (Thy1.2) mice depleted of endogenous Tregs, adoptive transfer of Tregs from donor CS-exposed B6.PL(Thy1.1) mice with subsequent *MTB* infection of the Thy1.2 mice resulted in a greater burden of *MTB* in the lungs and spleens than those that received Tregs from air-exposed mice. Mice that received Tregs from donor CS-exposed mice and infected with *MTB* had modest but significantly reduced numbers of interleukin-12-positive dendritic cells and interferon-gamma-positive CD4^+^ T cells in the lungs, and an increased number of total programmed cell death protein-1 (PD-1) positive CD4^+^ T cells in both the lungs and spleens.

**Discussion:**

Previous studies demonstrated that CS impairs macrophages and host-protective T effector cells in controlling *MTB* infection. We now show that CS-exposed Tregs can also impair control of *MTB* in co-culture with macrophages and in a murine model.

## Introduction

Worldwide, there are marked geographic overlaps between cigarette smoking and tuberculosis (TB) cases (Pai et al., 2007). Epidemiological studies show that individuals with cigarette smoke (CS) exposure have increased: infection rate with *Mycobacterium tuberculosis* (*MTB*), progression of primary infection, reactivation TB, severe disease, delay in sputum conversion, recurrence (relapse), worse treatment outcome, and TB mortality (Bishwakarma et al., 2015; Chiang and Bam, 2020; Wang et al., 2020). CS has deleterious effects on anti-TB immunity – including: *(i)* impairing macrophage killing of *MTB* by inhibiting phagosome-lysosome fusion, autophagy, efferocytosis, and apoptosis of infected macrophages, all due in part to polarization of macrophages to an immunosuppressive phenotype; *(ii)* decreasing the number of T_H_1 cells and production of interferon-gamma (IFNγ) from each cell; and *(iii)* increasing the expression of programmed death-ligand 1 and 2 (PD-L1/2) on antigen-presenting cells, which can lead to T effector cell exhaustion and death (Feng et al., 2011; Shang et al., 2011b; Shaler et al., 2013; Chan et al., 2014; O’Leary et al., 2014; van Zyl-Smit et al., 2014). However, the impact of CS on immunoregulating cells, particularly T regulatory cells (Tregs) has been less well studied (Bai et al., 2017). O’Leary et al (O’Leary et al., 2014) reported that both smokers’ and non-smokers’ alveolar macrophages could equally induce phenotypic differentiation of Tregs *ex vivo* but the co-culture of alveolar macrophages and naïve T cells were allogeneic and required stimulation with anti-CD3 antibody. Nevertheless, the findings from this previous study allude to the possibility that CS may impact immunosuppressive cells, leading to increased susceptibility to TB.

Murine studies have shown that knockdown of Tregs in the *early* phase of *MTB* infection results in reduced bacillus burden, indicating that an excessive number of Tregs soon after infection impairs host immunity against *MTB* (Ozeki et al., 2010). Moreover, active TB patients have higher Treg numbers than those with latent TB infection (Boer et al., 2015). Thus, a vigorous early influx of activated Tregs to the lungs, particularly before *MTB* infection is under control, worsens TB in experimental animals and is supported by congruent findings in primary human cells (Ordway et al., 2007; Shafiani et al., 2010; Shang et al., 2011a; Rowe et al., 2012; Larson et al., 2013; Semple et al., 2013).

Previously, our group and others showed that CS and nicotine could impair the ability of macrophages to control an *MTB* infection (Feng et al., 2011; Shang et al., 2011b; Shaler et al., 2013; Chan et al., 2014; O’Leary et al., 2014; van Zyl-Smit et al., 2014; Bai et al., 2017). However, since CS and nicotine may *(i)* increase the influx and function of Tregs in the lungs (Ménard and Rola-Pleszczynski, 1987; Barceló et al., 2008; Botelho et al., 2010; Wang et al., 2010), *(ii)* significantly increase the number of alveolar macrophages (Wallace et al., 1992), and *(iii)* alveolar macrophages of smokers can drive naïve T cells toward the Treg phenotype (O’Leary et al., 2014), it is plausible that CS could indirectly impact host immunity negatively against *MTB* by enhancing Treg activity. While the effects of CS on Treg number or activation in the context of *MTB* infection have not been previously reported, the effects of CS on Tregs have been described with somewhat contradictory findings (Qiu et al., 2017). On the one hand, several studies have shown that CS and nicotine may increase the number of Tregs and their activity in the lungs of both humans and mice (Ménard and Rola-Pleszczynski, 1987; Smyth et al., 2007; Barceló et al., 2008; Isajevs et al., 2009; Botelho et al., 2010; Wang et al., 2010; Shao et al., 2022). More specifically, CS led to an accumulation of FoxP3^+^ Tregs in the lungs of both C57BL/6 and BALB/c mice (Botelho et al., 2010) as well as in mouse spleens (Shao et al., 2022). In humans, active smokers – “healthy” individuals and those with chronic obstructive pulmonary disease – have increased numbers of Tregs in the lung lavage fluid or in the large airways compared with non- smokers (Smyth et al., 2007; Isajevs et al., 2009). On the other hand, others have also found that CS decreased Treg number or function albeit potential confounders for these alternative findings include: *(i)* the presence of underlying chronic obstructive pulmonary disease (in whom these studies are often done); *(ii)* the presence of cell types other than Tregs in the *in vitro* experiments (*e.g.*, CS extract decreased Treg numbers but only in co-culture with dendritic cells (Huang et al., 2021); and *(iii)* the differential effects of CS on different Treg subpopulations (Miyara et al., 2009; Qiu et al., 2017). This last confounder – *viz-a-viz* unique Treg subpopulations – underscores that the aforementioned discussions on Tregs and TB as well as CS and Tregs are oversimplified since at least three subpopulations of Tregs have been reported: *(i)* resting, suppressive Tregs (rTregs, CD25^+^FoxP3^lo^CD45RA^+^); *(ii)* activated, suppressive Tregs (aTregs, CD25^hi^FoxP3^hi^CD45RA^–^); and *(iii)* pro-inflammatory, cytokine-secreting (IFNγ and IL-2) Tregs (FrIII, CD25^+^FoxP3^lo^CD45RA^–^) (Miyara et al., 2009; Qiu et al., 2017).

To further elucidate the effects of CS on anti-TB host immunity, we undertook an *ex vivo* study of co-culture of human macrophages and air- or CS extract-exposed Tregs as well as an *in vivo* murine study using adoptive transfer of Tregs from air- or CS-exposed donor mice to determine whether CS-exposed Tregs could exacerbate *MTB* infection in the recipient mice.

## Materials and methods

### *Mycobacterium tuberculosis* strains, mice, and reagents

H37Rv-*MTB* was obtained from American Type Culture Collection (ATCC, Manassas, VA). HN878 W-Beijing strain of *MTB* (HN878-W-Beijing *MTB*) was originally a kind gift from Dr. B. Kreiswirth (Public Health Research Institute Center, Newark, NJ). The B6.PL(Thy1.1) and Foxp3^+^GFP^+^DTR^+^ (Thy1.2) mice were purchased from Jackson Laboratories, Bar Harbor, Maine. Cell Preparation Tubes (CPT) used to isolate peripheral blood mononuclear cells (PBMC) were purchased from BD Company (San Jose, CA). The Miltenyi Biotech Regulatory T Cell Isolation Kit II (human) (Auburn, CA) was used to isolate human Tregs from peripheral blood. 4′,6-diamidino-2-phenylindole (DAPI) and LysoTracker Red DND-99 were purchased from Invitrogen, Carlsbad, CA. Macrophage colony-stimulating factor (M-CSF, human) was purchased from Millipore Sigma, St. Louis, MO. Fetal bovine serum was acquired from Atlanta Biologicals (Norceross, GA) and inactivated at 56°C for one hour. The LC3-IIB and p62 primary rabbit antibodies were purchased from Cell Signaling Technology Inc. (Danvers, MA). Roswell Park Memorial Institute (RPMI) medium, CY3 goat anti-rabbit IgG (H+L) Cross-Adsorbed Secondary Antibody, and the four and eight chambered Nunc™ Lab-Tek™ II Chamber Slide™ System were purchased from ThermoFisher Scientific (Waltham, MA). PE-conjugated anti-CTLA-4 and APC-conjugated anti-CD279 (PD-1) were purchased from R&D System (Minneapolis, MN). Human cytokines from cell culture supernatants were analyzed using ELISA kits for TNF, IL-10, and TGFβ (R&D Systems, Minneapolis, MN and Invitrogen-ThermoFisher Scientific, Waltham, MA). Anti-CTLA-4 neutralizing antibody and non-immune human IgG antibody were purchased from BPS Bioscience Inc (San Diego, CA). Diphtheria toxin was purchased from List Biological Laboratories, Campbell, CA. Information on other reagents are discussed in the relevant methods sections below.

### Cigarette smoke extract preparation

CS extract was prepared by mechanically “smoking” a single unfiltered 3R4F cigarette into 10 mL of pre-warmed RPMI medium via a vacuum pump, with the flow adjusted at 3 L/min using an Accucal flowmeter (Gilmont Instruments). The extract was then filtered through a 0.22 µm syringe filter and adjusted to a pH of 7.4. This solution is arbitrarily designated as 100% CS extract (Ouyang et al., 2000).

### Blood processing and Treg isolation

After informed consent (Colorado Multiple Institutional Board Review, protocol 16-1413), blood was drawn into six Cell Preparation Tubes^®^ from each healthy donor. Peripheral blood mononuclear cells (PBMC) were isolated by centrifugation. Following the determination of the total cell count, half of the PBMC aliquot was cryopreserved in freeze medium (85% FBS, 15% DMSO) for future Treg isolation. The other half was incubated with 20 ng/mL of monocyte-colony stimulating factor for one week to prepare monocyte-derived macrophages (MDM).

One day before completion of MDM differentiation, an autologous cryopreserved PBMC aliquot was thawed and then centrifuged to remove the DMSO. Tregs were isolated from PBMC using the Regulatory T Cell Isolation Kit II according to manufacturer’s instructions. In brief, non-CD4^+^ and CD127^high^ cells were magnetically labeled and then depleted. From the remaining cells, CD25^+^ cells were magnetically labeled and then isolated, yielding only cells with biomarkers CD4^+^CD25^+^CD127^dim/-^, the cell surface signature of Tregs.

To confirm that Tregs were isolated, the cells were stained with CD127-BB515, CD25-PE, and CD4-APC-CY7, and analyzed with LSR Fortessa equipped with FACSDiva (BD Biosciences) and with the aid of FlowJo software. As shown in **Supplementary Figure 1**, essentially all the isolated cells had markers entirely consistent with Tregs.

### MDM infection and co-culture to quantify CFU

Differentiated MDM were infected with *MTB* H37Rv at a multiplicity-of-infection of 10 *MTB*:1 macrophage for one hour, and then washed gently to remove any free *MTB*. MDM were then incubated with: *(i)* RPMI medium + 10% FBS alone or with *(ii)* non-conditioned Tregs or *(iii)* Tregs previously conditioned in 5% CS extract for 18 to 24 hours at a ratio of 100 monocytes: 1 Treg. This ratio is the approximate ratio of lung macrophages:Tregs found in the bronchoalveolar lavage of healthy individuals (Heron et al., 2011). After an additional hour of incubation, Day 0 cells were washed, lysed, serially diluted, and plated on 7H10 agar to quantify *MTB*. Different aliquots of cells – incubated with *MTB* for one hour and then washed – were then incubated under the same three aforementioned conditions for 2 or 4 days before quantifying *MTB*.

### Phagosome-lysosome fusion

PBMC were differentiated into MDM in four-chamber glass slides. After one week, the MDM were co-cultured with autologous Tregs (100 MDM:1 Treg ratio) that had been previously cultured in medium alone or with 5% CS extract for 18 to 24 hours. MDM alone and MDM co-cultured with air- or CSE-exposed Tregs were infected with GFP-*MTB* H37Rv at a MOI of 10 *MTB*:1 macrophage for six hours. At the 4-hour mark, 25 µL of LysoTracker Red (50 nM) was added to each chamber well containing 500 µL of medium to label the lysosomes. At the 6-hour time point, the medium was removed, the cells fixed with 4% paraformaldehyde, washed thrice with 1X PBS, stained with DAPI and stored overnight to dry in the dark at room temperature. The following day, the cells were viewed under 40X oil immersion lens with an inverted Zeiss 200M microscope and a 175-watt xenon lamp in a DG4 Sutter instrument lamp housing. The Cy3 filter was used to detect the LysoTracker-stained lysosomes while FITC was used to detect GFP-*MTB*. Co-localization of the GFP-*MTB* with lysosomes would appear yellow. Ten random pictures were taken per well with Live-Cell Marianis. Images were further analyzed using FIJI software. Phagosome-lysosome (P-L) fusion was calculated by dividing the number of cells with evidence of GFP-*MTB* and lysosome fusion by the total number of cells containing GFP-*MTB*, the latter cell type comprised of at least 100 cells per condition (Bai et al., 2019).

### Autophagosome formation and maturation

MDM were co-cultured in four-chamber glass slides with Tregs (100 MDM:1 Treg) that were previously exposed to medium alone or 5% CS extract for 18 to 24 hours. After one hour of incubation with Tregs, the cells were infected with GFP-*MTB* (MOI of 10 *MTB*:1 macrophage) and incubated for 18 hours at 37°C and 5% ambient CO_2_. The cells were then washed with PBS, fixed with 4% paraformaldehyde for 30 minutes, washed, incubated with permeabilization buffer (0.5% Triton X in PBS) for 10 minutes, rinsed with PBS, and incubated with blocking buffer (5% BSA, 0.5% Tween 20 in PBS) for one hour. Anti-LC3 antibody at a dilution of 1.5:200 was incubated with the cells and left in a dark container at 4°C overnight. An anti-rabbit CY3 tagged antibody (1:1000) was added to the wells, left in the dark at room temperature for 45 minutes, and the cells were fixed with DAPI. The slides were dried overnight and viewed with the Live-Cell Marianis microscope to analyze and quantify autophagosome formation. Multiple images were taken from all conditions and five were picked at random. The number of LC3-II-positive puncta per cell was determined and averaged for 150 randomly chosen cells. The same process was replicated for all three conditions.

To determine autophagosome maturation, we quantified p62-positive puncta in a temporal fashion of MDM, MDM + unexposed Tregs, and MDM + CS extract-exposed Tregs that were infected with GFP-*MTB* for 3, 6, and 18 hours. At the end of each time point, the cells were washed with 1X PBS and fixed with 4% paraformaldehyde for 30 minutes. The cells were washed, permeabilized for 10 minutes, rinsed with 1X PBS, and incubated with a blocking buffer for one hour. Lastly, 25 µL of anti-p62-rabbit antibody [1:200] was added to each chamber well and incubated overnight at 4°C. Then CY3 anti-rabbit [1:1000] secondary antibody was added and incubated for 45 minutes in the dark. After incubation, slides were rinsed, DAPI added, and once dried, the cells were viewed under a 40X oil emersion lens. CY3 filter was used for detecting p62 and FITC for GFP-*MTB*. Further analyses of p62 in the presence of 200 nM rapamycin and 100 nM bafilomycin were conducted similarly.

### CS exposure of mice

The B6.PL(Thy1.1) mice were exposed to CS in a whole animal exposure chamber using smoke generated by the TE-10c cigarette smoking machine (Teague Enterprises) as previously described (Shang et al., 2011b). The relative proportion of smoke particulate, vapors, and gases generated approximate environmental CS exposure (89% side-stream smoke exposure and 11% mainstream CS exposure), maintaining a Total Suspended Particulate concentration of 85-120 mg/m^3^ for 5 hours a day and 5 days a week for four weeks.

### Depleting, harvesting, and adoptively transferring Tregs

Ten days before the adoptive transfer of Tregs, endogenous Tregs of the recipient Foxp3^+^GFP^+^DTR^+^ (Thy1.2) mice were depleted via administration of diphtheria toxin at 50 µg/kg x four doses intraperitoneally (Henao-Tamayo et al., 2016). Tregs of the air- and CS-exposed B6.PL(Thy1.1) mice were harvested by cell sorting. To sort CD4^+^CD25^hi^CD127^−^ and CD4^+^CD25^−^ cells, single cell suspension was prepared from the spleens of donor B6.PL(Thy 1.1) mice. After incubating with 5 µg/mL Fc block (ebioscience) for 20 min at 4°C, the cells were attained with APC labeled anti-CD4 (clone gk1.1; ebioscience), Pe Cy7-labeled anti-CD25 (clone PC61.5; ebioscience), Alexa ef450 labeled CD127 (eBioRDR5; ebioscience) and FITC-labeled anti-CD3 (clone 17A2; ebioscience). Thereafter, the cells were stained with 0.5 µg/ml 7-AAD (eBioscience) for 5 min, sorted on a FACS Aria III (BD Biosciences) with a 70-μm nozzle, doublets gated out by FSC-A vs. FSC-H, and 7-AAD^+^ dead cells excluded from the singlet population using FL3. The live CD4^+^CD25^hi^CD127^−^ or CD4^+^CD25^−^ were individually sorted. One million donor Tregs were transferred to each Treg-depleted Foxp3^+^GFP^+^DTR^+^ (Thy1.2) mouse via intravenous tail vein injection.

### Infection of Foxp3^+^GFP^+^DTR^+^ (Thy1.2) mice with *MTB*

Once the Foxp3^+^GFP^+^DTR^+^ (Thy1.2) mice received the Tregs from the donor B6.PL(Thy1.1) mice, the recipient mice were infected with HN878 W-Beijing strain of *MTB* using the Glas-Col Aerosol Generator at an inoculum dose of 2x10^6^ CFU/mL. The mice were exposed to an aerosol infection in which approximately 100 bacteria were deposited in the lungs of each mouse.

### *MTB* enumeration in mice

One, 30 and 60 days after infection, the mice were sacrificed by CO_2_ inhalation, and their lungs and spleen were prepared to quantify *MTB* (CFU) and immune cell phenotypes by flow cytometry and analyze the lungs by histopathology. The lungs and spleens were homogenized in saline and serial dilutions were plated on 7H11 agar plates supplemented with OADC (BD Biosciences, San Jose, CA). After 3-4 weeks of incubation at 37°C, CFUs were quantified and the data were expressed as the log_10_ numbers per target organ.

### Flow cytometry for surface markers and intracellular cytokines

For flow cytometric analysis, single-cell suspensions of lungs and spleen from each mouse were resuspended in PBS containing 0.1% sodium azide. Fc receptors were blocked with purified anti-mouse CD16/32. The cells were incubated in the dark for 25 minutes at 4°C with predetermined optimal titrations of specific antibodies. Cell surface expression was analyzed with antibodies for CD4 (clone GK1.5), CD11c (clone HL3), CD11b (clone M1/70), PD-1 (CD279) (clone J43), and CTLA-4 (clone 63828; R&D Systems). All antibodies and reagents were purchased from BD Pharmingen (San Diego, CA). The samples were probed using a Becton Dickinson FACSCanto instrument, and the data were analyzed using FlowJo software. Individual cell populations were identified according to the presence of specific fluorescence-labeled antibodies. A total of 300,000 gated events were collected for each sample and gated for each cell phenotype, differentiated by specific antibodies (Henao-Tamayo et al., 2010).

### Intracellular cytokine staining

The cells were initially stimulated for 4 hours at 37°C with a 1X cell stimulation cocktail (eBioscience) diluted in complete DMEM. Thereafter, the cells were stained for cell surface markers, fixed, and permeabilized according to the manufacturer’s instructions for the Fix/Perm and Perm wash kit (eBioscience). The cells were then incubated for 30 minutes at 4°C with FcBlock plus anti–IL-10 (clone JES5-16E3; eBioscience), anti–IFNγ (clone XMG1.2; eBioscience), anti-Foxp3 (clones FJK-16s; eBioscience), anti-IL-12 (clone C15.6; BD), or anti-TNF (clone MP6-XT22; eBioscience), or with the respective isotype control. Data acquisition was performed on FACSCanto cytometer (BD) and analysis was performed as described (Bai et al., 2015).

### Statistical analyses

Replicate experiments with duplicate wells for each condition per experiment are independent, and data are presented as means ± SEM or representative experiment. Group means were compared by repeated-measures ANOVA using Fisher’s least significant test or by two-way ANOVA with Bonferroni’s *post-hoc* test. Data were graphed in Prism 9^®^ and comparisons were considered significant when p<0.05.

## Results

### Cigarette smoke extract-exposed T regulatory cells further enhance proliferation of *Mycobacterium tuberculosis* in macrophages

**Figure 1:**
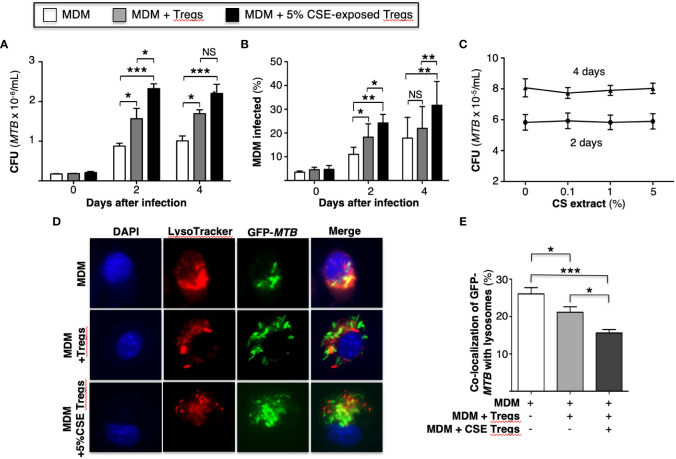
Cigarette smoke extract-exposed T regulatory cells impair macrophage control of *Mycobacterium tuberculosis* infection by reducing phagosome-lysosome fusion. **(A)** *MTB*-infected human monocyte-derived macrophages (MDM) alone or co-incubated with unexposed or 5% cigarette smoke (CS) extract-exposed T regulatory cells (Tregs) for the indicated times and intracellular *MTB* (CFU) quantified. **(B)** MDM were infected with green fluorescent protein (GFP)-*MTB* alone or co-incubated with unexposed or 5% CS extract-exposed Tregs, and at the indicated times, the percent of cells infected was calculated by dividing the number of GFP-*MTB* infected MDM by the total number of at least 500 MDM cells counted per condition. Data in **(A, B)** represent the mean ± SEM of experiments of cells performed in duplicates from three subjects. **(C)** *MTB* were cultured in 7H9 medium alone or with the indicated CS extract concentrations for 2 and 4 days and *MTB* quantified. Data in **(C)** represent the mean ± SEM of experiments of cells performed in duplicates of two experiments. **(D)** MDM were incubated in medium alone or co-cultured with unexposed or 5% CS extract-exposed Tregs, followed by infection with GFP-*MTB* and assayed for phagosome-lysosome (P-L) fusion. Shown are representative images of the identified conditions. **(E)** P-L fusion percentage was calculated by dividing the number of cells with evidence of P-L fusion by the total number of cells of five images (400X) for each condition. Data in **(D, E)** represent the mean ± SEM of four subjects, each with duplicate wells. *p<0.05, **p<0.01, and ***p<0.001. CFU=colony forming units; GFP=green fluorescent protein; *MTB*=*Mycobacterium tuberculosis*; NS=non-significant.

To determine whether CS extract-exposed human Tregs impair macrophage control of *MTB*, MDM were first infected with *MTB* for 1 hour, washed to removed free *MTB*, and then co-cultured with autologous primary human Tregs (at a ratio of 100 monocytes: 1 Treg (Heron et al., 2011)) that were previously incubated in medium alone or with 5% CS extract for 18-24 hours. It is important to emphasize that the Tregs were washed to remove any medium containing CS extract before co-culture with the MDM to ensure that any effect of CS extract was caused by the CS extract-exposed Tregs and not due to any direct effects of CS extract on MDM. After the *MTB*-infected cell co-cultures were incubated for 1 hour, 2 and 4 days, cell-associated *MTB* were quantified as previously reported (Bai et al., 2017). As an additional control, MDM without Tregs were also infected with *MTB*. Compared to MDM alone, co-culture of MDM with control Tregs increased the bacterial burden, which further increased in co-culture with CS extract-exposed Tregs (**Figure 1A**).

An alternative approach to assess bacterial burden was employed in which MDM alone, MDM + unexposed Tregs, and MDM + CS extract-exposed Tregs were infected with GFP-*MTB* H37Rv for 1 hour, 2 and 4 days and the percentage of GFP-*MTB*-infected MDM determined by fluorescent microscopy. Compared to MDM alone, co-culture of Tregs with MDM increased the percentage of MDM containing GFP-labeled *MTB*, which further increased with the addition of Tregs that were pre-incubated with CS extract (**Figure 1B**). CS extract itself did not directly enhance the growth of *MTB* in the absence of macrophages (**Figure 1C**). We conclude that CS extract-exposed Tregs further enhance proliferation of *MTB* in macrophages.

### Cigarette smoke extract-exposed T regulatory cells inhibit phagosome-lysosome fusion in *Mycobacterium tuberculosis*-infected macrophages

An immune evasive mechanism of *MTB* is the inhibition of phagosome-lysosome (P-L) fusion. To determine whether CS extract-exposed Tregs affect P-L fusion in *MTB*-infected macrophages, MDM were cultured alone or co-cultured with autologous Tregs previously exposed to medium alone or with 5% CS extract for 18-24 hours; this was followed by infection with GFP-*MTB* H37Rv for 6 hours and quantitation of P-L fusion. Compared to GFP-*MTB*-infected MDM alone, co-incubation of MDM with Tregs reduced the co-localization of GFP-*MTB* with lysosomes, with even further decrease when MDM were co-cultured with CS extract-exposed Tregs (**Figures 1D, E**).

### Cigarette smoke extract-exposed T regulatory cells inhibit autophagosome formation and maturation of *Mycobacterium tuberculosis*-infected macrophages

**Figure 2:**
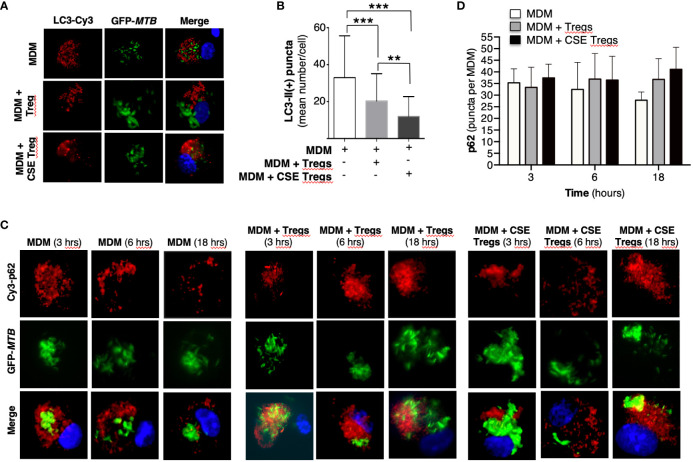
Cigarette smoke extract-exposed T regulatory cells decreases autophagosome formation. **(A)** Monocyte-derived macrophages (MDM) were incubated in medium alone or co-cultured with unexposed or cigarette smoke (CS) extract-exposed T regulatory cells (Tregs), followed by infection with green fluorescent protein-labeled *Mycobacterium tuberculosis* (GFP-*MTB*) for 18 hours and assayed for LC3-II expression by immunofluorescence. **(B)** Mean LC3-II positive puncta were calculated by counting the individual puncta of *MTB*-infected MDM (150 cells per condition). **(C)** MDM were incubated in medium alone or co-cultured with unexposed or 5% CS extract-exposed Tregs, followed by infection with GFP-*MTB* for 3, 6 and 18 hours, and assayed for p62 expression by immunofluorescence. Data represent the mean ± SEM of four independent experiments. **(D)** Mean p62(+) puncta were calculated by counting the individual puncta of *MTB*-infected MDM (50 cells per condition). Data represent the mean ± SEM of three independent experiments, each with duplicate wells. **p<0.01 and ***p<0.001.

To quantify autophagosome formation, MDM, MDM + unexposed Tregs, and MDM + CS extract-exposed Tregs infected with *MTB* for 18 hours were immunostained with anti-LC3-II antibody and Cy3-tagged goat anti-rabbit antibody. MDM co-incubated with CS extract-exposed Tregs had significantly fewer LC3-II (+) puncta than MDM alone or MDM + unexposed Tregs (**Figures 2A, B**). These findings indicate that in the presence of CS extract-exposed Tregs, *MTB*-infected MDM had either decreased autophagosome formation, increased autophagosome-lysosome fusion with degradation of autophagosome content, or both.

To analyze which of these two autophagic processes is more dominant in MDM + CS extract-exposed Tregs, expression of the cargo protein sequestosome-1 (p62) – located within autophagosomes and degraded upon fusion with lysosomes – was temporally quantified. MDM, MDM + unexposed Tregs, and MDM + CS extract-exposed Tregs infected with *MTB* for 3, 6, and 18 hours were immunostained for p62. With MDM alone, there was a modest decrease in p62(+) autophagosomes with increasing time of infection, suggesting increased autophagosome-lysosome fusion (**Figures 2C, D**, open bars). However, in the presence of either unexposed Tregs or CS extract-exposed Tregs, the number of p62(+) autophagosomes did not decrease but there was a modest increase, suggesting a component of blockade in autophagosome-lysosome fusion in the presence of Tregs. Examination of the 18-hour time point shows a modest stepwise increase in p62(+) autophagosomes with MDM, MDM + unexposed Tregs, and MDM + CS extract-exposed Tregs (**Figure 2D**, last three bars), indicating decreased autophagosome-lysosome fusion in the presence of Tregs, especially those that were exposed to CS extract.

**Figure 3:**
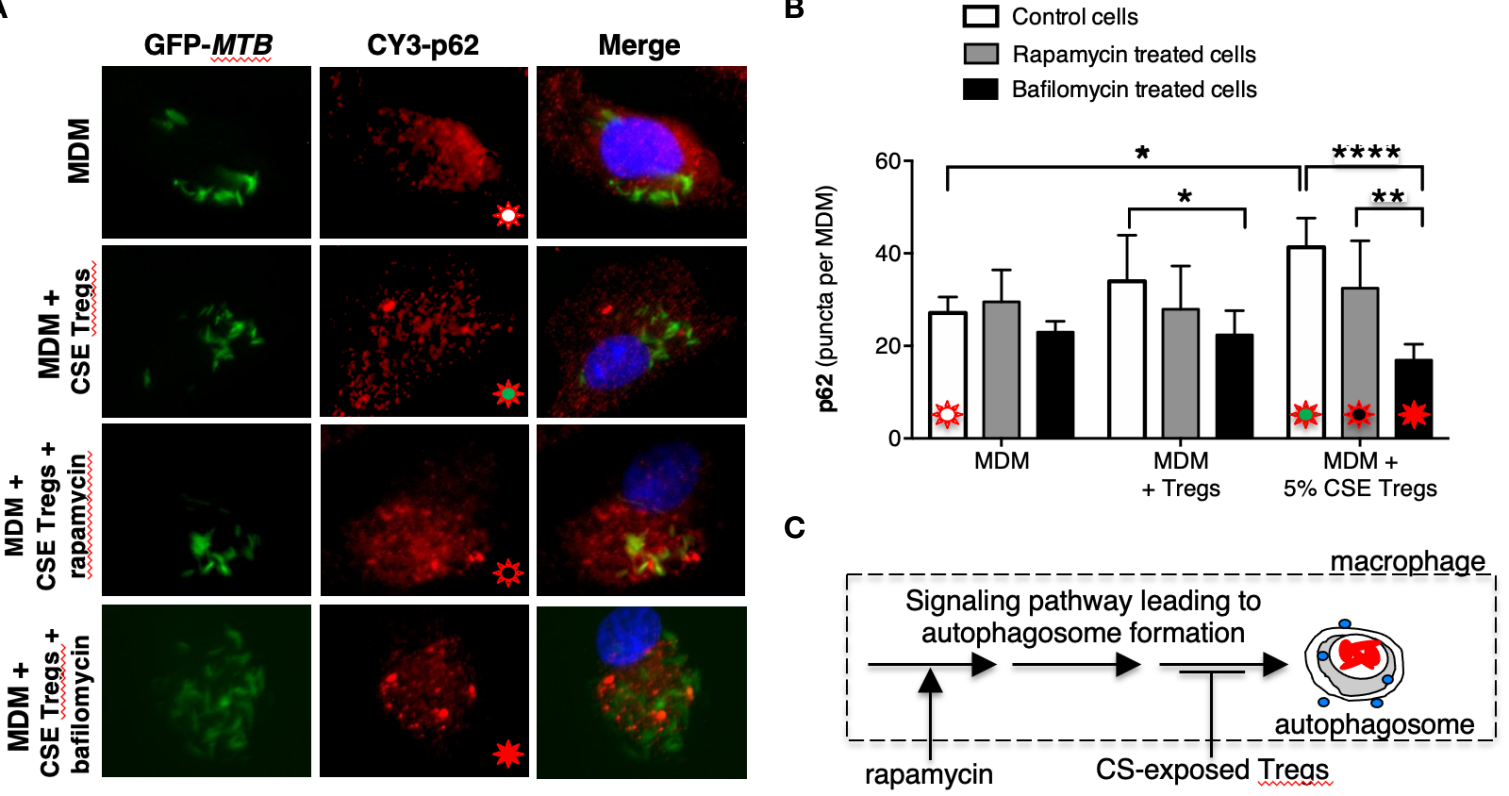
Cigarette smoke extract-exposed T regulatory cells decreases autophagosome formation at a site distal to rapamycin induction of autophagosomes. **(A)** Monocyte-derived macrophages (MDM) were incubated in medium alone or co-cultured with unexposed Tregs or cigarette smoke (CS) extract-exposed Tregs ± rapamycin or bafilomycin [100 nM] for 18 hours, followed by quantifying p62 expression by immunofluorescence. **(B)** Mean p62 puncta for a total of 50 cells per condition for the indicated conditions. **(C)** Based on the results, we hypothesize that CS extract inhibits autophagy at a site distal to where rapamycin induces autophagosome formation. Data represent the mean ± SEM of three independent experiments, each with duplicate wells. The four different colored starbursts in the representative crucial immunofluorescent photomicrographs in **(A)** correspond to those shown in the bar graphs in **(B)**. *p<0.05, **p<0.01, and ****p<0.0001.

To further validate these findings, we quantified p62(+) puncta in the MDM, MDM + unexposed Tregs, and MDM + CS extract-exposed Tregs at 18 hours after infection with *MTB* in the presence of rapamycin, a known inducer of autophagy (Ko et al., 2017). As previously shown, there was a successive increase in p62 puncta with MDM alone, MDM + unexposed Tregs, and MDM + CS extract-exposed Tregs (**Figure 3B**, open bars). In contrast, in MDM plus either unexposed or CS extract-exposed Tregs treated with rapamycin, there was no increase in p62(+) puncta compared to cells not treated with rapamycin (**Figure 3B**, compare gray bars vs. respective open bars). Because rapamycin did not increase the number of autophagosomes in co-culture of MDM + CS extract-exposed Tregs, it suggests CS extract-exposed Tregs are inhibiting the signaling pathway that leads to autophagosome formation at a site “downstream” to where rapamycin is augmenting autophagosome formation (**Figure 3C**).

To determine whether CS extract-exposed Tregs are inducing autophagosome-lysosome fusion (= autophagosome maturation) to account for the decreased LC3-II(+) autophagosomes, we performed the aforementioned p62 assay in the absence or presence of bafilomycin, which inhibits both lysosome acidification and autophagosome-lysosome fusion (Mauvezin and Neufeld, 2015). We hypothesized that if CS extract-exposed Tregs were inducing autophagosome-lysosome fusion, then in the presence of bafilomycin, we would expect a relative increase in the number of p62(+) puncta because bafilomycin would be inhibiting the degradation of p62(+) autophagosomes. Unexpectedly, addition of bafilomycin decreased the number of p62(+) puncta, especially in MDM + 5% CS extract-exposed Tregs (**Figures 3A, B**, last open vs. black bars). However, the size of the p62(+) puncta with bafilomycin treatment were noticeably larger than seen in MDM without bafilomycin treatment, suggesting autophagosome aggregation (**Figure 3A**, **Supplementary Figure 2A**). Using FIJI software, the p62(+) puncta were measured by area and fluorescence using a Corrected Total Cell Fluorescence equation provided by Luke Hammond from The University of Queensland, Australia (Hammond, 2014). These analyses showed that the total area fluorescence of the p62(+) puncta were not significantly different with or without bafilomycin (**Supplementary Figures 2B, C**). These findings support the paradigm that co-incubation of MDM with CS-exposed Tregs reduces autophagosome number by inhibiting their formation (as noted by decreased LC3-II autophagosomes) but also a component of decreased autophagosome maturation (as noted by increased p62(+) marker to the autophagosomes).

### Cytokine expression in *Mycobacterium tuberculosis*-infected macrophages with or without co-culture with unexposed and cigarette smoke extract-exposed T regulatory cells

**Figure 4:**
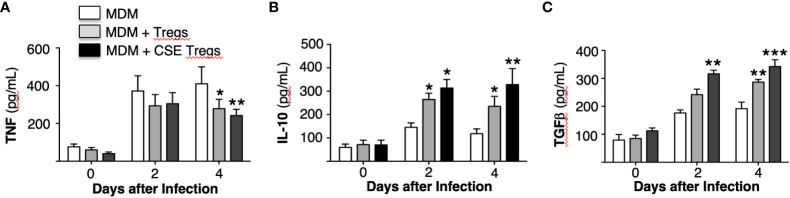
Cytokine expression by *Mycobacterium tuberculosis*-infected macrophages ± unexposed or cigarette smoke extract-exposed T regulatory cells. Monocyte-derived macrophages (MDM) and T regulatory cells (Tregs) were prepared from four different donors. Unexposed or cigarette smoke (CS) extract-exposed Tregs were added to MDM infected with *MTB*. These co-cultures and MDM alone infected with *MTB* were cultured for 1 hour, 2 and 4 days and the supernatants were obtained and measured for **(A)** TNF, **(B)** IL-10, and **(C)** TGFβ. Data shown are mean ± SEM of four independent experiments with each performed in duplicate wells. *p<0.05, **p<0.01, ***p<0.001. *MTB*=*Mycobacterium tuberculosis*.

The supernatant of the MDM, MDM + unexposed Tregs, and MDM + CS extract-exposed Tregs used to obtain the aforementioned *MTB* CFU were saved and assayed for tumor necrosis factor (TNF), interleukin-10 (IL-10), and transforming growth factor-beta (TGFβ). Compared to MDM alone, co-culture with unexposed Tregs modestly decreased TNF and increased IL-10 and TGFβ at 2 and 4 days after culture (**Figures 4A-C**). Co-culture of MDM with Tregs exposed to CS extract further decreased TNF level and increased levels of IL-10 and TGFβ (**Figures 4A-C**).

### Cigarette smoke extract induction of CTLA-4 on T regulatory cells impairs macrophage control of *Mycobacterium tuberculosis* infection

**Figure 5:**
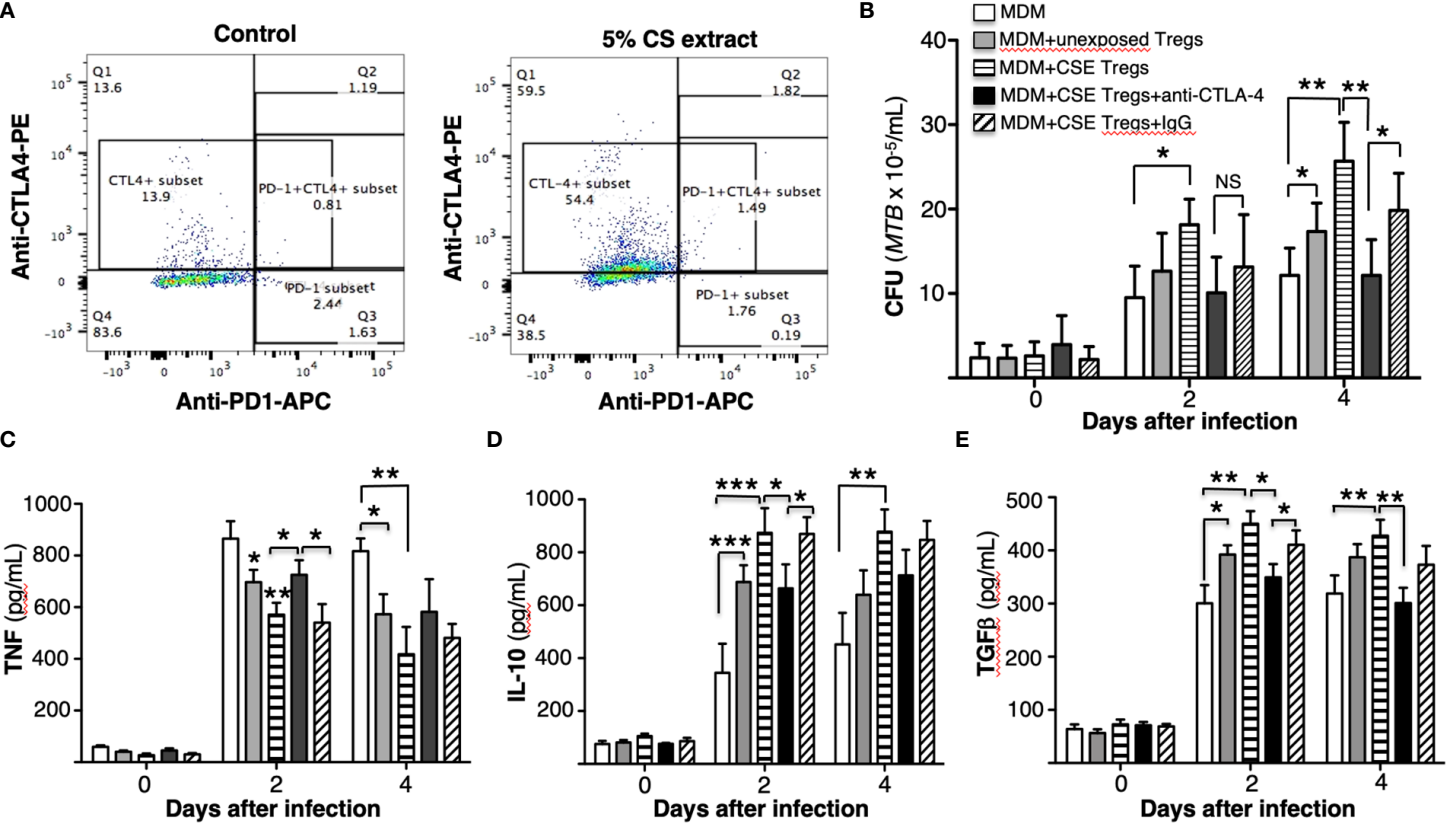
Cigarette smoke extract induction of CTLA-4 in T regulatory cells increases their ability to impair macrophage control of *Mycobacterium tuberculosis*. **(A)** Unexposed or cigarette smoke (CS) extract-exposed T regulatory cells (Tregs) of a healthy subject were stained with PE-conjugated anti-CTLA-4 and APC-conjugated anti-CD279 (PD-1) and flow cytometry was performed. **(B)** Monocyte-derived macrophages (MDM) infected with *MTB* were cultured alone or co-cultured with unexposed or CS extract-exposed Tregs ± anti-CTLA-4 or non-immune IgG for 1 hour, 2, and 4 days. After the indicated times, intracellular *MTB* was quantified. The mean ± SEM of cells derived from four different donors and performed in duplicates are shown. **(C-E)** Human MDM and T regulatory cells (Tregs) were prepared from three different donors. MDM infected with *MTB* were cultured alone or co-cultured with unexposed or CS extract-exposed Tregs ± anti-CTLA-4 or non-immune IgG for 1 hour, 2, and 4 days and the supernatants were obtained and measured for **(C)** TNF, **(D)** IL-10, and **(E)** TGFβ. Data shown are mean ± SEM of three independent experiments with each performed in duplicate wells. *p<0.05, **p<0.01, ***p<0.001. *MTB*=*Mycobacterium tuberculosis*; NS=non-significant.

In human lungs, CS exposure promotes Treg function (Ménard and Rola-Pleszczynski, 1987; Barceló et al., 2008; Wang et al., 2010). A plausible mechanism by which this occurs is the ability of CS to induce PD-L1/2 on antigen presenting cells, resulting in increased engagement to PD-1 on Tregs (Francisco et al., 2010; Yanagita et al., 2012); *i.e.*, whereas engagement of PD-1 and cytotoxic T-lymphocyte-associated protein 4 (CTLA-4) on T cells to PD-L1/2 and B7 on macrophages, respectively, inhibits the activity of Foxp3-negative T effector cells, these same molecular interactions enhance the suppressive activity of Tregs (Saresella et al., 2008; Chan et al., 2014). Thus, to determine whether PD-1 and/or CTLA-4 plays a mechanistic role by which CS extract-exposed Tregs impair macrophage control of *MTB* infection, we first exposed Tregs to CS extract for 18 hours and quantified PD-1 and CTLA-4 expression. CS extract increased the expression of CTLA-4 but not PD-1 in primary human Tregs (**Figure 5A**).

To determine the role CS extract-induced CTLA-4 on Tregs may play in impairing MDM control of *MTB*, we incubated CS extract-exposed Tregs with 5 µg/mL non-immune IgG or with 5 µg/mL anti-CTLA-4 antibody before combining the Tregs with *MTB*-infected MDM (Machicote et al., 2018). As shown in **Figure 5B**, macrophages co-cultured with CS extract-exposed Tregs have greater *MTB* burden than MDM alone or MDM + unexposed Tregs. However, in the presence of an anti-CTLA-4 neutralizing antibody, there was a partial but significant abrogation of the increase in *MTB* burden in co-culture of MDM + CS extract-exposed Tregs; such abrogation was not seen with non-immune IgG. These findings indicate that CS extract induction of CTLA-4 on Tregs is a mechanism by which CS extract-exposed Tregs impair macrophage control of *MTB* infection.

The supernatant of the MDM, MDM + unexposed Tregs, MDM + CS extract-exposed Tregs, and MDM + CS extract-exposed Tregs incubated with IgG or anti-CTLA-4 (and all infected with *MTB*) were assayed for TNF, IL-10, and TGFβ. As previously seen, compared to MDM alone, co-culture with unexposed Tregs modestly decreased TNF and increased IL-10 and TGFβ at 2 and 4 days after culture (**Figures 5C-E**). Co-culture of MDM with Tregs exposed to CS extract further decreased TNF level and increased IL-10 and TGFβ. Compared to MDM + CS extract-exposed Tregs, neutralization of CTLA-4 significantly abrogated the reduction in TNF at Day 2 after *MTB* infection (**Figure 5C**) and abrogated the increase in IL-10 at Day 2 and TGFβ at Day 4 after *MTB* infection (**Figures 5D, E**).

### Adoptive transfer of T regulatory cells from cigarette smoke-exposed mice increased *Mycobacterium tuberculosis* burden in recipient mice

**Figure 6:**
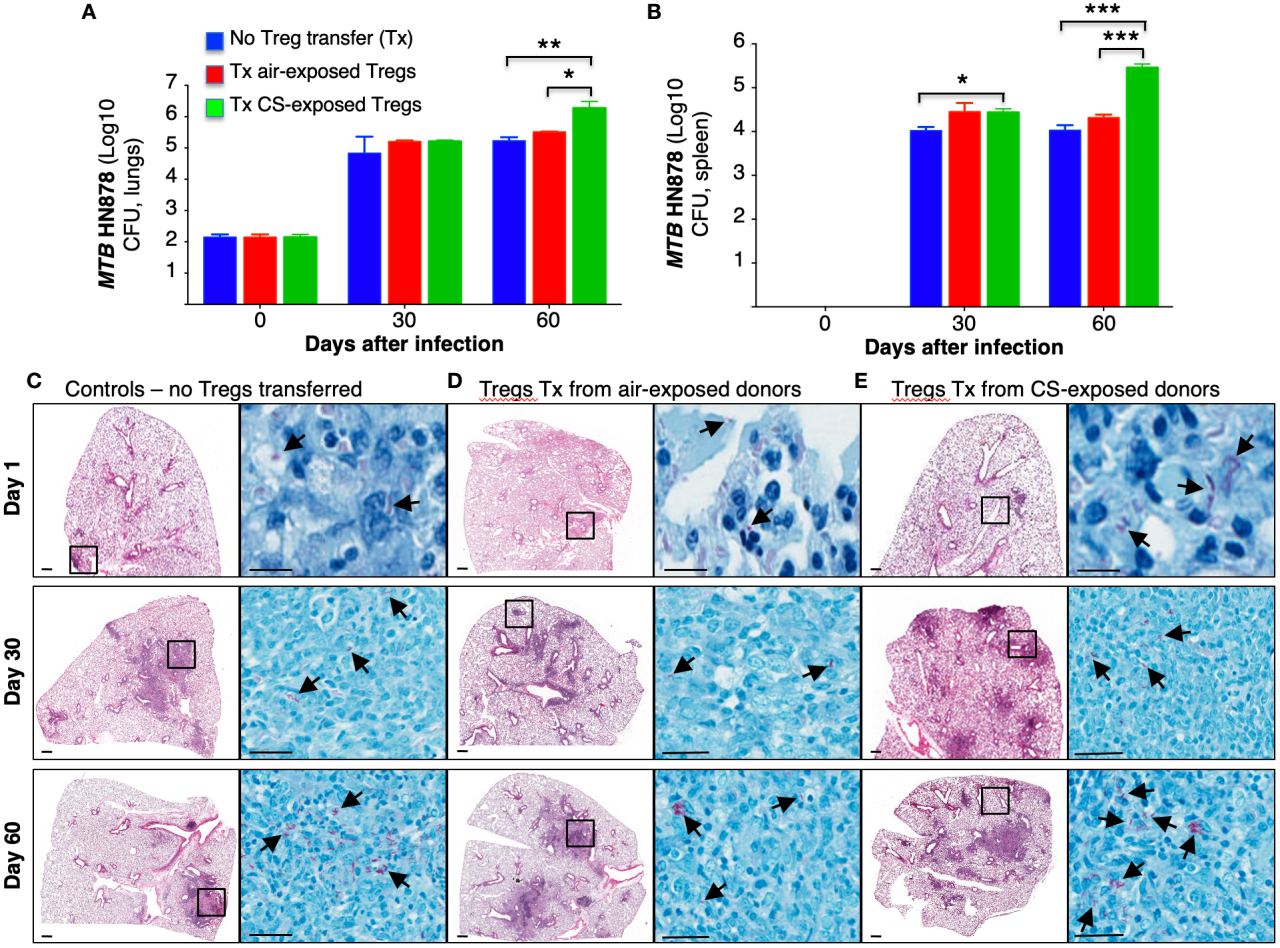
Mice receiving T regulatory cells from cigarette smoke-exposed mice have greater burden of *Mycobacterium tuberculosis* in the lungs and spleen. **(A)** T regulatory cells (Tregs) were isolated from two groups of Thy1.1 mice; one group was exposed to cigarette smoke (CS), while the other was exposed to ambient air. The isolated Tregs from unexposed and CS-exposed Thy1.1 mice were adoptively transferred into the recipient, Treg-depleted Thy1.2 mice. A third group of control mice did not undergo Treg depletion or adoptive transfer. All three groups of mice were then infected with *MTB* HN878. The mice from each group were sacrificed at the one, 30 and 60 days after infection. The lungs of mice were homogenized to quantify CFU. **(B)** The spleens of mice were also homogenized to determine CFU. Representative histopathologic photomicrograph (H&E and acid-fast stains) are shown of **(C)** control mice that were infected with *MTB* alone and recipient mice that had previously received Tregs from **(D)** air- or **(E)** CS-exposed mice and then infected with *MTB*. Data shown are the mean ± SEM of cells derived from five mice. *p<0.05, **p<0.01, ***p<0.001. *MTB*=*Mycobacterium tuberculosis*.

Tregs in the Foxp3^+^GFP^+^DTR^+^ (Thy1.2) mice were depleted by administration of four doses of diphtheria toxin (DT) as we previously reported, wherein ~6-fold depletion of Tregs were achieved in the lungs and >10-fold depletion of Tregs in the spleen (Henao-Tamayo et al., 2016). In this study, we used higher amounts of DT for the last two doses. Following the adoptive transfer of Tregs from air-exposed and CS-exposed B6.PL(Thy1.1) donor mice into Treg depleted Foxp3^+^GFP^+^DTR^+^ (Thy1.2) mice, the latter recipient mice were infected with HN878-W-Beijing *MTB* for 1, 30, and 60 days. Because these mouse strains possess an immunocompetent phenotype, a hypervirulent strain of *MTB* was chosen to achieve a productive infection. Foxp3^+^GFP^+^DTR^+^ (Thy1.2) mice in which no Treg depletion or transfer was performed were also infected with HN878 *MTB* as an additional control. The lungs and spleen were harvested, processed, and the CFU of the organs were determined at the indicated times. Compared to control mice and mice that received Tregs from air-exposed mice, those that received Tregs from CS-exposed mice had significantly greater CFU in the lungs and spleens at Day 60 after infection (**Figures 6A, B**).

### Lung histopathology of mice that received T regulatory cells from cigarette smoke-exposed mice revealed larger, less-discrete lung lesions

Histopathology of the lungs was analyzed at 1, 30 and 60 days after HN878 *MTB* infection. Compared to control mice and mice recipient of Tregs from air-exposed donors, mice that received Tregs from CS-exposed donors demonstrated increased size and number of granuloma-like lesions, increased granulocyte infiltration, and the presence of acid-fast bacilli, particularly in the lesions (**Figures 6C–E**, arrows).

### Immunophenotyping of murine lung macrophages and dendritic cells

**Figure 7:**
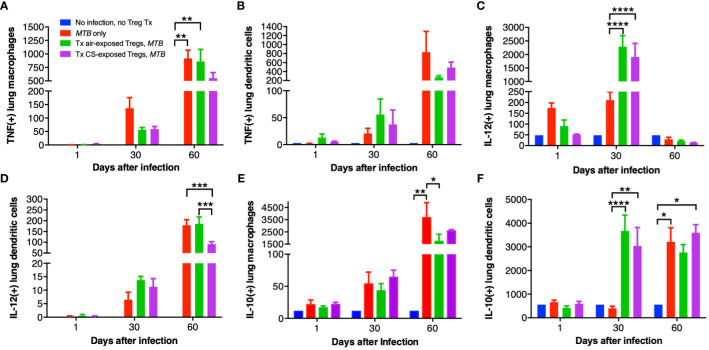
Intracellular cytokine analysis of murine lung macrophages and DCs. Lung macrophages and dendritic cells from uninfected mice, *MTB*-infected mice, and *MTB*-infected mice with adoptive transfer of T regulatory cells (Tregs) from either air-exposed or CS-exposed mice were stained for **(A/B)** TNF, **(C/D)** IL-12, and **(E/F)** IL-10. Data shown are the mean ± SEM of cells derived from five mice. For the “no infection, no Treg transfer” condition, there was only one mouse used. *p<0.05, **p<0.01, ***p<0.001, ****p<0.0001. *MTB*=*Mycobacterium tuberculosis*.

Lung macrophages and dendritic cells (DC) of uninfected mice, control *MTB*-infected mice, and Foxp3^+^GFP^+^DTR^+^ (Thy1.2) mice that received air- or CS-exposed Tregs followed by *MTB* infection were immunophenotyped for intracellular TNF, IL-12, and IL-10 at 1, 30 and 60 days after *MTB* infection. Compared to no infection, *MTB* infection increased the number of TNF^+^ lung macrophages and DC at 30 and 60 days after infection (**Figures 7A, B**). At 60 days after infection, there was decreased number of TNF^+^ macrophages in mice transferred Tregs from CS-exposed mice although this did not reach statistical significance (**Figure 7A**). At 60 days post infection, there was also a trend toward decreased TNF^+^ DC in mice transferred Tregs from either air- or CS-exposed mice (**Figure 7B**).

Compared to uninfected mice, *MTB* infection alone increased the number of IL-12^+^ lung macrophages, which further increased with the transfer of either air- or CS-exposed Tregs at 30 days post-infection (**Figure 7C**). Interestingly, at 60 days post-infection, IL-12^+^ lung macrophages decreased to levels even lower than at 1 day after infection. In contrast, IL-12^+^ DC progressively increased temporally with all three *MTB*-infected conditions (**Figure 7D**); in mice that received Tregs from CS-exposed mice, there was a significantly lower number of IL-12^+^ DC compared to uninfected mice, mice only infected with *MTB*, or mice that received Tregs from air-exposed mice and then infected with *MTB* (**Figure 7D**).

There was a temporal increase of IL-10^+^ lung macrophages and DC with *MTB* infection (except for DC at Day 30 after infection) (**Figures 7E, F**). Mice that received Tregs from either air- or CS-exposed mice have decreased IL-10^+^ macrophages at Day 60 compared to *MTB*-infected mice without Treg transfer (**Figure 7E**). In contrast, mice that received either air- or CS-exposed Tregs and *MTB*-infected have marked infiltration with IL-10^+^ DC at Day 30 compared to mice infected with *MTB* only, but this difference was lost at Day 60 (**Figure 7F**).

**Figure 8:**
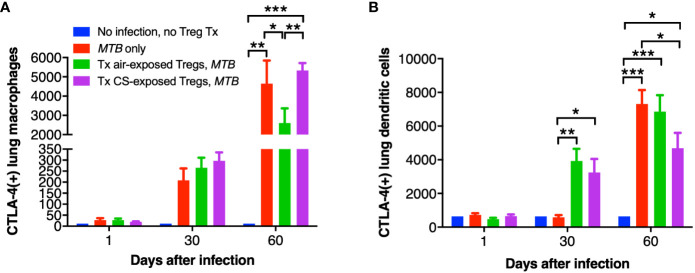
CTLA-4 expression in the lung macrophages and dendritic cells of the recipient mice. At the indicated times, uninfected mice, after *MTB*-infected Thy1.2 mice, and mice that received T regulatory cells (Tregs) from either air- or cigarette smoke (CS)-exposed Thy1.1 mice and subsequently infected with *MTB* were sacrificed and CTLA-4^+^ lung **(A)** macrophages and **(B)** dendritic cells were quantified by flow cytometry. Data shown are the mean ± SEM of cells derived from five mice. For the “no infection, no Treg transfer” condition, there was only one mouse used. *p<0.05, **p<0.01, ***p<0.001. *MTB*=*Mycobacterium tuberculosis*.

While CTLA-4 is canonically expressed on T cells, it is also present on human monocytes and mature monocyte-derived DC following stimulation with lipopolysaccharide, poly:IC, and various cytokines; CTLA-4 on DC also inhibits CD4^+^ T cell proliferation via CTLA-4-mediated production of IL-10 by the DC (Laurent et al., 2010). Thus, we quantified CTLA-4 expression in the mouse lungs and found that with *MTB* infection, there was a temporal increase in CTLA-4^+^ macrophages with *MTB* infection with or without Treg transfer (**Figure 8A**). However, in the mice that received air-exposed Tregs, there was a lower amount of CTLA-4^+^ macrophages at Day 60, but in the mice that received Tregs from CS-exposed mice, there was higher number of CTLA-4^+^ macrophages (**Figure 8A**). There was also a temporal increase of CTLA-4^+^ DC with all infections – except for Day 30 with the only *MTB*-infected mice (**Figure 8B**). In contrast to that seen with lung macrophages, there was a significant decrease of CTLA-4^+^ DC in the mice that received Tregs from CS-exposed mice at Day 60 compared to mice with *MTB* infection alone. We conclude that in mice that received Tregs from CS-exposed mice, there was greater decrease in the number of TNF^+^ macrophages, IL-12^+^ DC, and CTLA-4^+^ DC in the lungs compared to that seen in control mice that either received no Tregs or air-exposed Tregs.

### Immunotyping of murine lung CD4^+^ T cells

**Figure 9:**
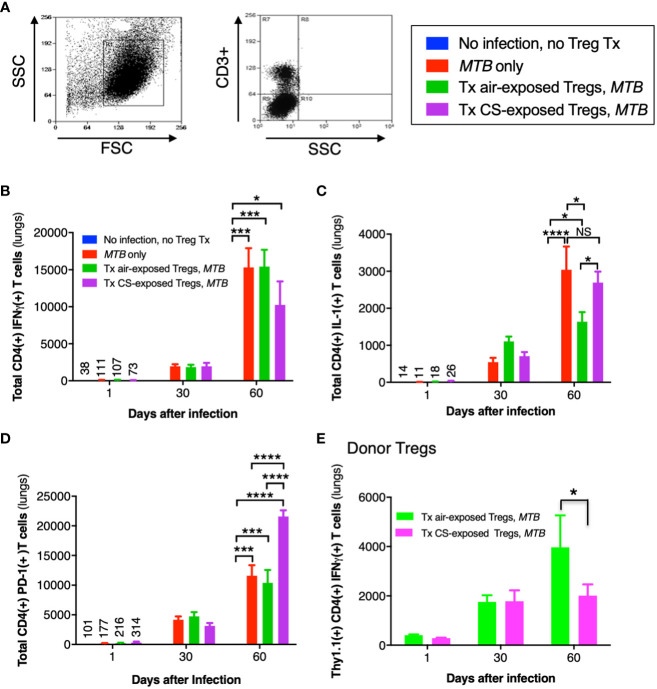
Cytokine expression of T cells in the lungs of mice that received adoptive transfer of T regulatory cells. **(A)** General gating strategy for T cells in the murine lungs. **(B)** CD4^+^IFNγ^+^ T cells, **(C)** CD4^+^IL-1^+^ T cells, and **(D)** CD4^+^PD-1^+^ T cells were quantified in the lungs of the uninfected mice, *MTB*-infected mice, and Thy1.2 mice that received T regulatory cells (Tregs) from air- or cigarette smoke (CS)-exposed Thy1.1 mice and then infected with *MTB*. **(E)** Thy1.1 CD4^+^IFNγ^+^ transferred Tregs quantified in the lungs of the recipient Thy1.2 mice at the indicated times. Data shown are the mean ± SEM of cells derived from five mice. For the “no infection, no Treg transfer” condition, there was only one mouse used. *p<0.05, ***p<0.001, and ****p<0.0001. *MTB*=*Mycobacterium tuberculosis*.

The general gating strategy for T cells is shown in **Figure 9A**. Examining the phenotypic subsets of T cells revealed a temporal increase in CD4^+^IFNγ^+^ T cells in the lungs of all three mouse groups infected with *MTB* compared to uninfected mice (**Figure 9B**). But at Day 60, the number of CD4^+^IFNγ^+^ T cells in mice that received Tregs from CS-exposed mice and infected with *MTB* was less than all other mouse groups but these differences were not statistically significant (**Figure 9B**, last bar vs. preceding two bars at Day 60).

Compared to uninfected mice, the number of CD4^+^IL-1^+^ T cells increased temporally in the other mouse groups infected with *MTB* (**Figure 9C**). While the number of CD4^+^IL-1^+^ T cells were similar at Days 1 and 30 between all three groups of *MTB*-infected mice, by Day 60 the mice that received air-exposed Tregs had the least number of IL-1^+^CD4^+^ T cells (**Figure 9C**). In examining an exhaustion marker of CD4^+^ T cells, mice that received Tregs from CS-exposed, *MTB*-infected mice had significantly more PD-1-positive T cells at Day 60 (**Figure 9D**, last bar vs. preceding two bars at Day 60). However, there was no difference in the expression of either PD-1 or CTLA-4 donor Thy1.1^+^Tregs from unexposed or CS-exposed mice in the lungs of the recipient mice at 1, 30, or 60 days after infection (data not shown).

Quantifying the total number of CD4^+^ Tregs in the four groups of mice (uninfected, *MTB*-infected alone, and *MTB*-infected mice recipient of Tregs from air- or CS-exposed mice), we found that compared to uninfected mice, *MTB*-infected mice had significantly more total Tregs, which peaked at Day 30 after infection (**Supplementary Figure 3A**). While there was a lower number of total CD4^+^ Tregs at Day 30 after infection in the mice that received Tregs from CS-exposed mice, this was not statistically significant. Similarly, there was a trend of increased number of CD4^+^IL-10^+^ Tregs at Day 60 after *MTB* infection in the mice that received Tregs from either air- or CS-exposed mice (**Supplementary Figure 3B**). Since a small population of Tregs can produce IFNγ (Zhao et al., 2011; Koenecke et al., 2012; Sumida et al., 2018), we analyzed the population of transferred Thy1.1^+^CD4^+^IFNγ^+^ Tregs from the air- or CS-exposed Thy1.1 mice in the lungs of the recipient Thy1.2 mice following *MTB* infection. While there was no difference in the population of these Tregs in the lungs at Day 1 or 30, by Day 60 the recipient Thy1.2 mice had fewer donor Thy1.1^+^IFNγ^+^ Tregs from the CS-exposed mice compared to that from the air-exposed mice (**Figure 9E**). Furthermore, the temporal increase in the donor CD4^+^IFNγ^+^ Tregs indicates replication of these donor Tregs in the recipient mice over the course of the infection as previously reported (Henao-Tamayo et al., 2016).

### Immunophenotyping of murine splenic macrophages, dendritic cells, and T cells

Splenic macrophages and DC were also analyzed 2, 30, and 60 days after *MTB* infection in the three mouse groups. Uninfected mice also served as an additional control. Compared to uninfected mice, the three *MTB*-infected mouse groups demonstrated an increase in intracellular TNF^+^, IL-12^+^, and IL-10^+^ macrophages (**Supplementary Figures 4A-C**) and DC (**Supplementary Figure 4D-F**). With the time points examined, the number of such macrophage and DC phenotypes were highest at 60 days after *MTB* infection except for the IL-12^+^ macrophages, which peaked at Day 30 (**Supplementary Figure 4B**). Similarly, the three mouse groups infected with *MTB* had similar numbers of CTLA-4^+^ macrophages (**Supplementary Figure 4G**) and dendritic cells (**Supplementary Figure 4H**), with both cell type numbers highest at 60 days after infection.

Analysis of the total number of splenic CD4^+^ T cell subsets revealed that, compared to uninfected mice, the number of T cells significantly increased at 30 and 60 days in the three mouse groups that were infected with *MTB* (**Supplementary Figure 5**). More specifically, the total number of IFNγ^+^ T cells increased progressively from Day 2 to Day 60 after infection in control mice that were only infected with *MTB* and in *MTB*-infected mice that received Tregs from air-exposed mice. In contrast, the temporal increase in the number of IFNγ^+^ T cells was attenuated in the animals that received Tregs from CS-exposed mice (**Supplementary Figure 5A** **, last bar vs. preceding two bars at Day 60**). Similarly, compared to uninfected mice, there was an increase in the number of IL-1^+^ T cells in the three mouse groups infected with *MTB* (**Supplementary Figure 5B**). The number of such IL-1^+^ T cells peaked at Day 30 in the mice infected with *MTB* alone and mice that received Tregs from air-exposed mice whereas they peaked at Day 60 in the mice that received Tregs from CS-exposed mice and subsequently infected with *MTB* (**Supplementary Figure 5B**). In examining the CD4^+^ T cell subset expressing the PD-1 exhaustion marker, there was a progressive temporal increase in all three groups of mice infected with *MTB* (**Supplementary Figure 5C**). However, there was significantly greater number of PD-1^+^ splenic T cells in the *MTB*-infected mice that received Tregs from CS-exposed mice (**Supplementary Figure 5C**).

In analyzing the splenic Tregs, there was an increased in the total number at Days 30 and 60 in the three mouse groups infected with *MTB* compared to uninfected mice (**Supplementary Figure 5D**). Compared to *MTB* infection alone, those that received air-exposed mice had a robust rise in Tregs at Day 30, which decreased to near basal levels at Day 60. In contrast, there was a progressive increase in Tregs in mice that received Tregs from CS-exposed mice. The number of IL-10^+^ Tregs temporally quantified in the spleen followed a very similar pattern to that observed with the total number of Tregs (**Supplementary Figure 5E**).

## Discussion

While robust epidemiologic evidence shows that CS exposure is significantly associated with the development of active TB and with greater severity (Bishwakarma et al., 2015), less is known of the mechanisms by which this link occurs. CS has been shown to polarize macrophages toward the M2/deactivated phenotype and to impair macrophage control of *MTB* (Shaykhiev et al., 2009; Shang et al., 2011b; O’Leary et al., 2014; van Zyl-Smit et al., 2014). Previous studies of mice exposed to CS and subsequently infected with *MTB* demonstrated similarly decreased influx of host-protective cells, particularly IFNγ-producing T_H_1 cells and M1 type macrophages, and an increased number of CD4^+^IL-4^+^ T cells (Feng et al., 2011; Shang et al., 2011b; Shaler et al., 2013). However, less is even known about how CS may affect the immunosuppressive cells such as Tregs in the context of TB.

Tregs are specialized CD4^+^ T cells that develop from naïve T cells in the presence of TFGβ. In turn, Tregs produce anti-inflammatory cytokines TGFβ, IL-35 and IL-10 (Vignali et al., 2008; Shevach, 2009; Cavassani et al., 2010) although a subset of Tregs (FrIII) is known to secrete IFNγ and IL-2 (Miyara et al., 2009; Qiu et al., 2017). Through the anti-inflammatory cytokines, Tregs can suppress anti-TB immunity during the early stages of infection (Ordway et al., 2007; Shafiani et al., 2010; Shang et al., 2011a; Larson et al., 2013; Semple et al., 2013). Another immunosuppressive mechanism of Tregs is their ability to inhibit T effector cells, partly through the secretion of granzyme B, which breaches the cell walls of T effector cells, causing them to undergo apoptosis in a perforin-dependent fashion (Grossman et al., 2004). Tregs may also suppress M1 macrophage activation and induce macrophage differentiation to the M2 phenotype (Tiemessen et al., 2007; Martinez et al., 2009; Shevach, 2009). M2 macrophages exhibit impaired induction of pro-inflammatory cytokines and macrophage effector functions against *MTB* such as P-L fusion, autophagy, and apoptosis (Mills, 2012; Romano et al., 2018). In addition, the cell surface protein LAG3 on Tregs binds to MHCII on DC, preventing further DC activation (Liang et al., 2008). The surface protein TIGIT found on T effector cells and Tregs also binds DC causing secretion of TGFβ and IL-10, immunosuppressive cytokines capable of inhibiting T effector cells and DC (Yu et al., 2009). However, Tregs may also be necessary at the latter stages of infection when *MTB* comes under control to prevent inflammatory tissue damage that may occur, with the injury contributed by an excessive response of CD4^+^IFNγ^+^ T cells (Kumar, 2017; Cardona and Cardona, 2019).

CS and nicotine are also known to increase the activation or production of Tregs (Ménard and Rola-Pleszczynski, 1987; Barceló et al., 2008; Botelho et al., 2010; Wang et al., 2010). Using primary murine cells, we previously demonstrated that nicotine-exposed Tregs impaired murine macrophages from controlling an *ex vivo MTB* infection (Bai et al., 2017). This current study revealed that CS exposure of *only* primary human Tregs followed by their co-culture with unexposed macrophages enhances proliferation of *MTB* in the macrophages by inhibiting both P-L fusion and autophagosome formation/maturation.

While rapamycin is known to increase autophagosome formation by inhibiting mTOR, it could also indirectly drive autophagosome degradation – analogous to LeChatelier’s principle with chemical reactions by providing an increased number of the substrate (autophagosomes). Thus, if rapamycin-induced autophagosome degradation rate is greater than rapamycin-induced autophagosome formation rate and combined with decreased autophagosome formation due to the CS extract-exposed Tregs, one may not see an increase in autophagosomes with rapamycin and in fact may see a modest decrease, which is what was observed (**Figures 3A, B**). These pathways are made more complex by the finding that rapamycin can also increase p62 mRNA and protein expression in an autophagy independent pathway (Ko et al., 2017). We also found that in the presence of bafilomycin, an inhibitor of autophagosome-lysosome fusion, there was no significant difference in the total p62(+) puncta, which would be an expected finding if an increase in p62(+) puncta due to bafilomycin was countered by decreased autophagosome formation from the CS extract-exposed Tregs. We conclude from these autophagy experiments that CS extract-exposed Tregs reduce autophagosome formation in macrophages and that this reduction prevailed over the decrease in autophagosome maturation.

One molecular mechanism for the increased Treg activity with CS or nicotine exposure is induction of CTLA-4 and PD-L1, the latter a ligand for PD-1 (Zhang and Petro, 1996; Wing et al., 2008; Francisco et al., 2010; Yanagita et al., 2012). While engagements of CTLA-4 and PD-1 to their respective ligands deactivate effector T cells, they enhance Treg function, as supported by the observation that blockade of CTLA-4 and PD-1 are known to exacerbate autoimmune diseases through deactivation of Tregs (Walker, 2013; Kong and Flynn, 2014). But in human Tregs, we did not find an increase in the number of PD-1 positive Tregs but rather an increase in CTLA-4 positive Tregs with CS extract exposure. We further showed that CS induction of CTLA-4 on Tregs is a mechanism by which CS-exposed Tregs impair macrophage control of *MTB* infection.

The murine adoptive transfer studies corroborated the *in vitro* human cell data in that recipient Thy1.2 mice that received Tregs from donor CS-exposed mice had increased *MTB* burden in both the lungs and spleens compared to the two murine controls (*MTB* infection of naïve mice and of mice that received Tregs from air-exposed mice). Interestingly, the increase in CFU of the mice that received Tregs from CS-exposed mice was not observed until Day 60 in the lungs and spleens. While there was a significant increase in CFU with mice that received Tregs from CS-exposed mice in the spleen at Day 30, it was by ~one-half log of CFU increase.

The increased *MTB* burden in the lungs and spleens of the mice that received Tregs from CS-exposed mice was not due to increased number of Tregs (**Supplementary Figure 3A**), indicating conceivably that CS exposure of Tregs increased their immunosuppressive activity, consistent with prior studies showing that CS and nicotine may increase the influx and function of Tregs in the lungs (Ménard and Rola-Pleszczynski, 1987; Barceló et al., 2008; Botelho et al., 2010; Wang et al., 2010). Hence, compared to the two *MTB*-infected mouse control groups, the increased CFU seen in mice that received Tregs from CS-exposed mice may be due to the following immunophenotypic findings: significant decrease in IL-12^+^ DC, an increase in CTLA-4^+^ macrophages, and an increase in CD4^+^PD-1^+^ T cells and a trend toward increased IL-10^+^ macrophages and decreased TNF^+^ lung macrophages, IL-12^+^ macrophages, and total IFNγ^+^CD4^+^ T cells and Thy1.1^+^CD4^+^IFNγ^+^ transferred Tregs. However, in recipient mice that received Tregs from donor CS-exposed mice, we found fewer CTLA-4^+^ DC. CTLA-4 has been previously reported on macrophages (Hua et al., 1999; Wang et al., 2002) and dendritic cells (Wang et al., 2011) and may play a role in inhibiting immune response. Thus, this finding seems paradoxical since fewer CTLA-4^+^ DC would be expected not to inhibit CD4^+^ T effector cell as much, and thus would have relatively greater host immunity against *MTB* (Laurent et al., 2010).

The observed reduction in IFNγ^+^ T cells in mice that received Tregs from CS-exposed donor mice would be expected to reduce macrophage activation and MHCII expression. Since a subset of Tregs can also produce IFNγ (Miyara et al., 2009; Zhao et al., 2011; Koenecke et al., 2012; Sumida et al., 2018), it is interesting to note that mice that received Tregs from either air- or CS-exposed mice had similar number of donor (Thy1.1) IFNγ^+^ Tregs at Days 1 and 30 after infection, and yet mice that received Tregs from CS-exposed mice had decreased number of donor IFNγ^+^ Tregs at Day 60 compared to those that received Tregs from air-exposed mice (**Figure 9E**). While we did not directly examine the three subpopulations of Tregs, this finding would suggest that CS exposure – in a delayed fashion and following *MTB* infection – resulted in a decrease in the number of pro-inflammatory, cytokine-producing (FrIII) Treg subpopulation (Miyara et al., 2009). In a recent review of Tregs and *MTB* infection, no mention was made regarding the three subpopulations of Tregs although they alluded to another type of activated, immunosuppressive Tregs (CD4^+^CD25^hi^CD39^+^) (Cardona and Cardona, 2019). Mice that received air-exposed Tregs had fewer CD4^+^IL-1^+^ T cells than those that received Tregs from CS-exposed mice; this finding was somewhat unexpected since IL-1 is generally considered host-protective.

There are several limitations to this study although we do not believe they detract from the key finding that CS-exposed Tregs impair host-immunity against *MTB*. Firstly, cytokine levels were not measured in unexposed and CS extract-exposed human Tregs alone, which would help determine the contribution of Tregs vs. MDM in cytokine expression in response to CS. However, because Tregs are not known to phagocytose *MTB*, the significance of cytokine expression of CS-exposed Tregs alone (in the absence of *MTB*-infected macrophages) would be difficult to interpret. Secondly, since we did not see a difference in PD-1 or CTLA-4 expression of the donor Thy1.1^+^ Tregs in the lungs of the recipient mice, another limitation of the study is that we did not quantify Tim3 expression in these Tregs as Tim3 is increased in smokers and is increasingly recognized as an exhaustion marker for T effector cells (Valiathan et al., 2014) and thus could be an activation marker for Tregs – as has been described for PD-1 and CTLA-4. Thirdly, we did not examine separately the three subpopulations of Tregs in either the human co-culture with macrophages or the murine adoptive transfer studies; however, such approaches would be extremely challenging technically with the likely possibility that not enough Tregs of each subpopulation would be able to be isolated for the required experiments. Fourthly, we used CS extract for the *ex vivo* human cell experiments and whole CS for the *in vivo* studies. While both human cell and murine adoptive studies showed similar relative CFU findings with CS extract- vs. whole CS-exposed Tregs, some differences were observed in the ancillary findings; *e.g.*, whereas unexposed Tregs + macrophages and especially CS-exposed Tregs + macrophages produced significantly more IL-10 than macrophages alone in the human cell studies (**Figures 4B**, **5D**), there was a decrease in IL-10^+^ macrophages at Day 60 after infection in mice that received Tregs from either unexposed or CS-exposed donor mice (**Figure 7E**). While such differences may be due to multiple factors, one possible reason is the difference in CS product used in the human cell (CS extract) and murine adoptive transfer (whole CS) studies. Fifthly, we used two different strains of *MTB*, H37Rv-*MTB* for the *ex vivo* human cell experiments and *HN878-W-Beijing MTB* for the *in vivo* mouse investigations. Thus, it would be interesting to compare laboratory and clinical *MTB* strains in future studies for each set of experiments.

In conclusion, we found that CS extract-exposed Tregs or Tregs from CS-exposed mice plays an active role in impairing human macrophage and mouse immunity, respectively, against *MTB*. Mice that received Tregs from CS exposed mice were also more compromised in controlling *MTB* infection due mainly to a decrease in both host-protective effector innate and T cell phenotypes. In primary human cells, CS extract-exposed Tregs impaired macrophage control of *MTB* by inhibiting P-L fusion and autophagosome formation and to a lesser extent by inhibiting autophagosome maturation.

## Data availability statement

The original contributions presented in the study are included in the article/**Supplementary Material**. Further inquiries can be directed to the corresponding authors.

## Ethics statement

The studies involving humans were approved by COMIRB (University of Colorado Anschutz Medical Campus) and IRB (National Jewish Health) for Human Subjects. The studies were conducted in accordance with the local legislation and institutional requirements. The participants provided their written informed consent to participate in this study. The animal study was approved by ACORP (Rocky Mountain Regional VA Medical Center) & IACUC (National Jewish Health) for Animals. The study was conducted in accordance with the local legislation and institutional requirements.

## Author contributions

XB, DO, and EC conceived the study. XB, DV, LL, NW, JG, TO, and DO conducted the murine experiments and analyzed the data. CG, AM, KK, LF, ME, and MG were responsible for the human cell studies. All authors contributed to the intellectual input, writing, editing, and approving the manuscript.
