# Supplementary material for: Ex vivo and in vivo evidence that cigarette smoke-exposed T regulatory cells impair host immunity against Mycobacterium tuberculosis
Source: Front Cell Infect Microbiol. 2023 Oct 26;13:1216492. doi: 10.3389/fcimb.2023.1216492 (PMC10641287; doi:10.3389/fcimb.2023.1216492)
Supplement: Supplementary file 1 [file Image_1.pdf]

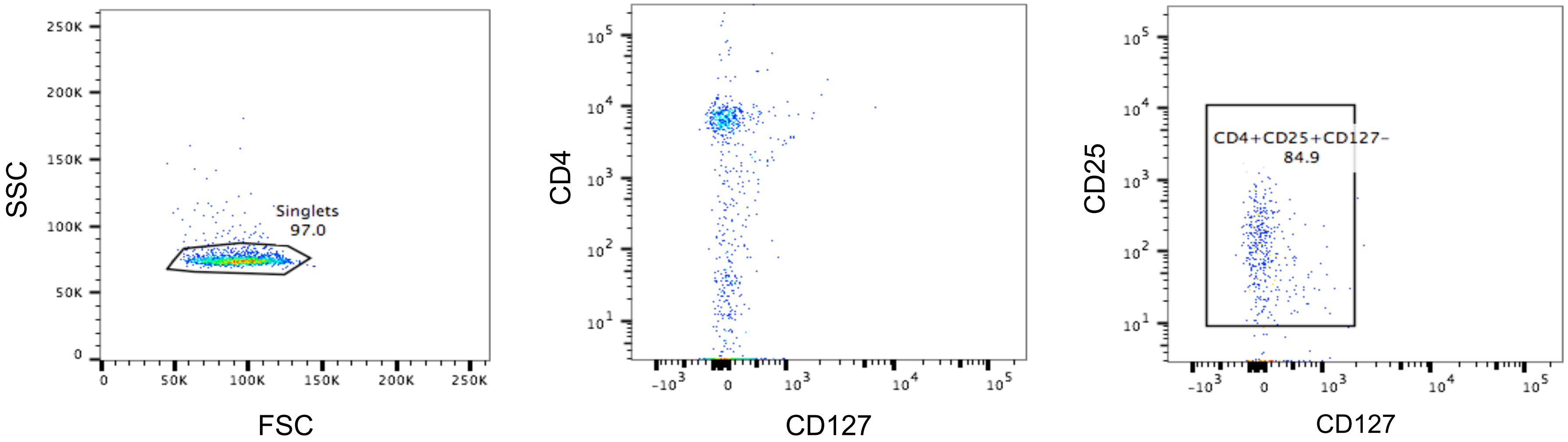

Supplemental Figure 1. Human T regulatory cell isolation. T regulatory cells (Tregs) were isolated from peripheral blood mononuclear cells using Miltenyi Biotec's Human Treg Isolation Kit II. Following isolation, the cells were stained for CD127-BB515, CD25-PE, and CD4-APC-CY7 to confirm Treg enrichment by flow cytometry.

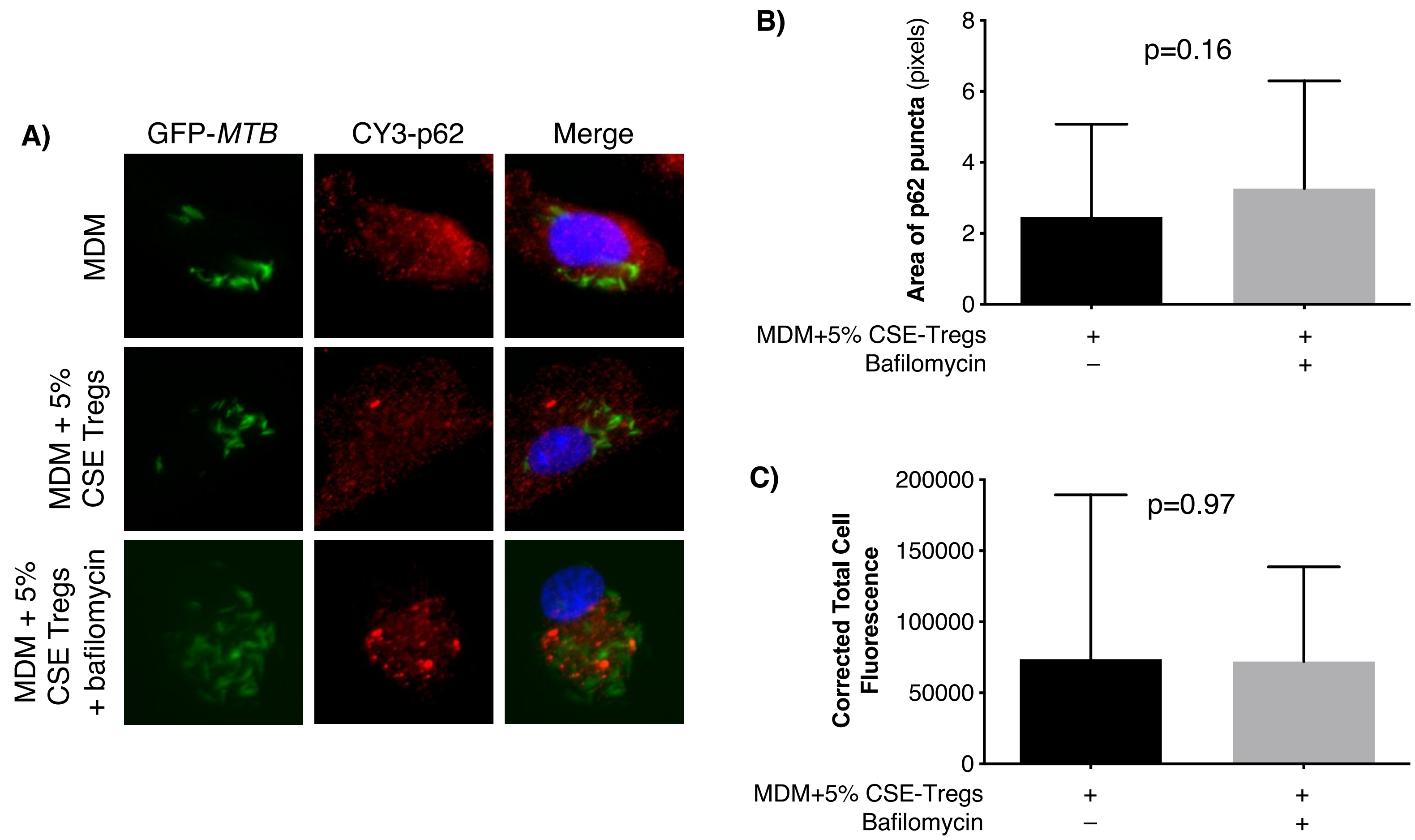

Supplemental Figure 2. Puncta size and corrected total cell fluorescence of bafilomycin treated cells. (A) Human monocyte-derived macrophages (MDM) were incubated as previously stated, followed by infection with a 10:1 MOI of GFP-*MTB* with or without bafilomycin for 18 hours and assayed for p62 expression immunofluorescence. **(B)** The area of p62 puncta was measured using FIJI software. A total of 300 puncta were measured, the graph depicts the mean area of the p62(+) puncta in MDM co-cultured with cigarette smoke (CS) extract-exposed Tregs without or with bafilomycin. **(C)** Corrected total cell fluorescence (CTCF) was calculated using the FIJI Software, which calculates the integrated density of an image selected (in this case, an MDM). The area of the MDM is multiplied by the mean fluorescence of the background image (an area of the image around the MDM). This number is then subtracted from the integrated density previously calculated. The equation was contributed by The QBI Advanced Microscopy facility, The University of Queensland, Australia. GFP-*MTB*=green fluorescent protein-labeled *Mycobacterium tuberculosis*.

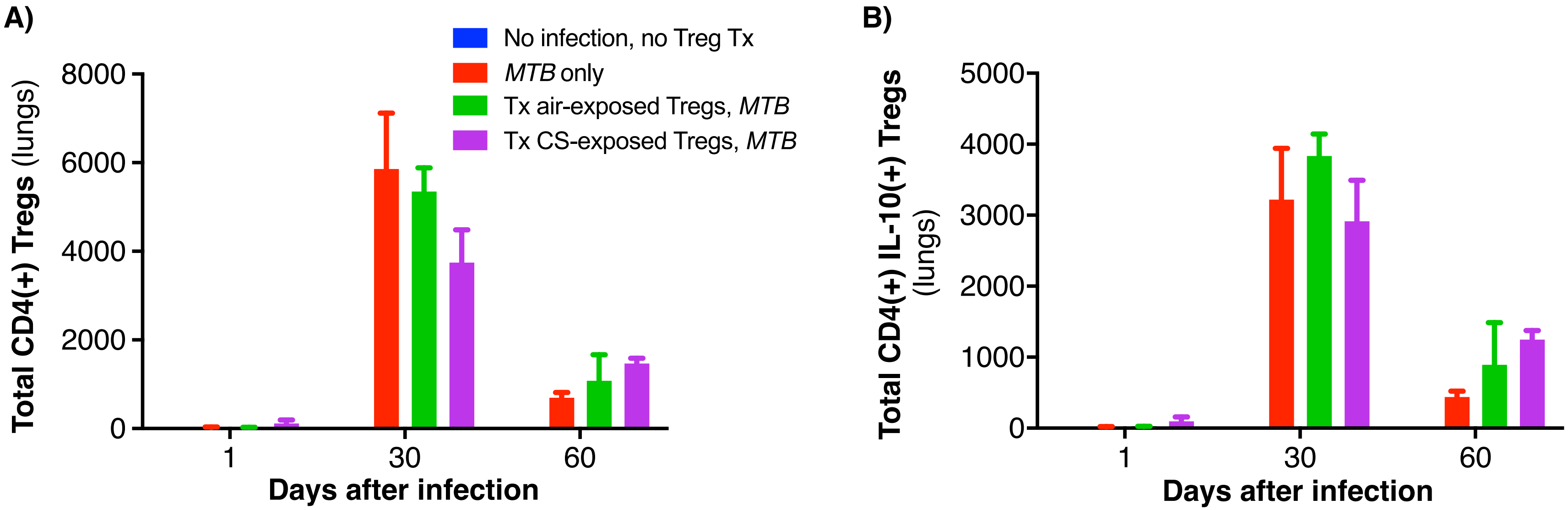

Supplemental Figure 3. Quantitation of T regulatory cells from the lungs of mice that underwent adoptive transfer. (A) Total $CD4^{+}CD25^{hi}CD127^{-}$ (T regulatory cells) were quantified in the lungs of uninfected mice, *MTB*-infected mice, and Thy1.2 mice that received T regulatory cells (Tregs) from air- or cigarette smoke (CS)-exposed Thy1.1 mice and then infected with *MTB*. **(B)** Total $CD4^{+}CD25^{hi}CD127^{-}IL\text{-}10^{+}$ in the lungs of the same mouse groups were quantified. *MTB=Mycobacterium tuberculosis.*

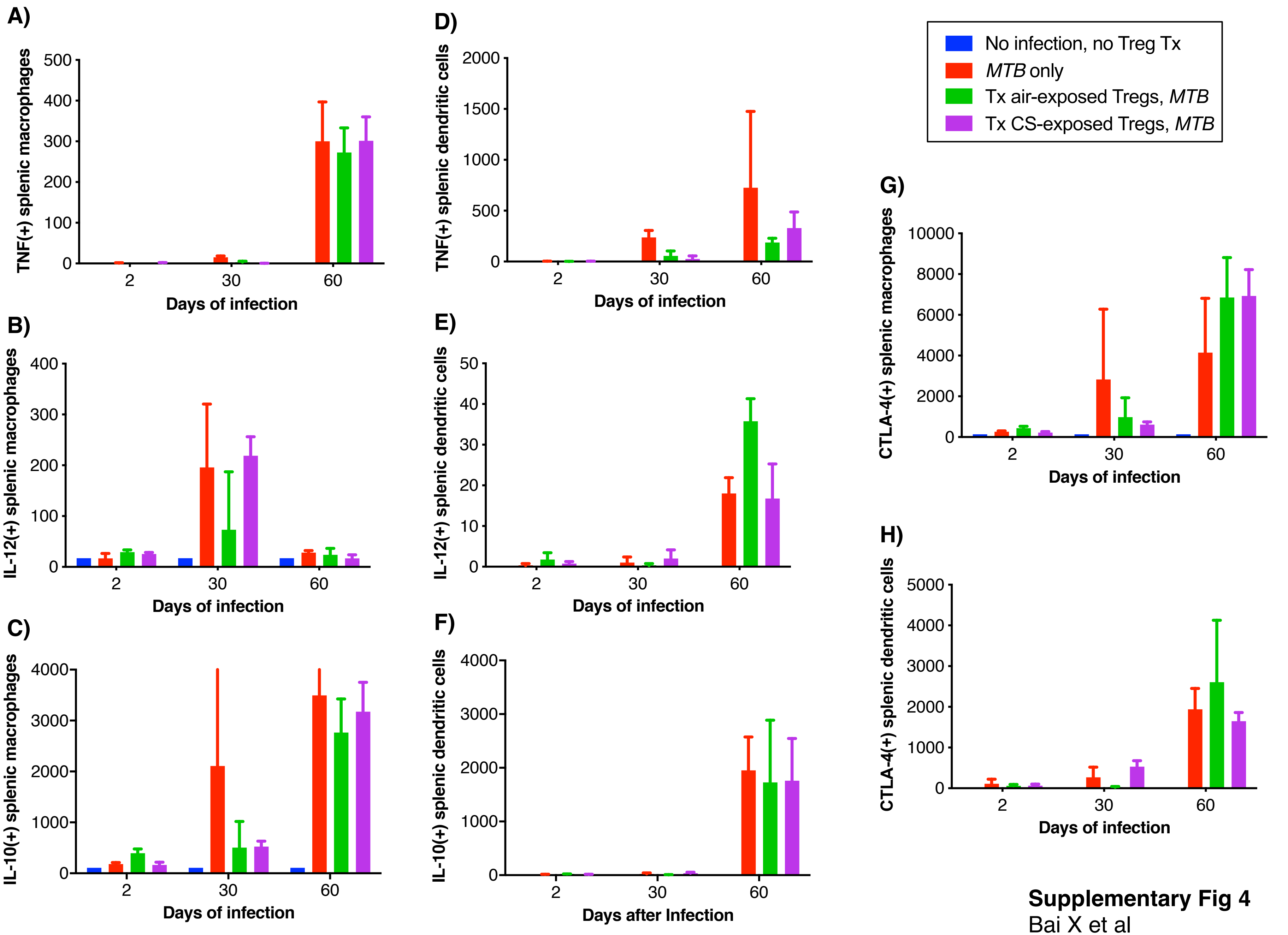

Supplementary Fig 4
Bai X et al

Supplemental Figure 4. Intracellular cytokine and CTLA-4 analyses of murine splenic macrophages and dendritic cells. Splenic macrophages and dendritic cells from uninfected mice, *MTB*-infected mice, and *MTB*-infected mice with adoptive transfer of Tregs from either air-exposed or CS-exposed mice were stained for **(A/D)** TNF, **(B/E)** IL-12, and **(C/F)** IL-10. From the same mouse groups, splenic macrophages and dendritic cells were stained for cell surface CTLA-4 **(G/H)**, respectively. *MTB*=*Mycobacterium tuberculosis.*

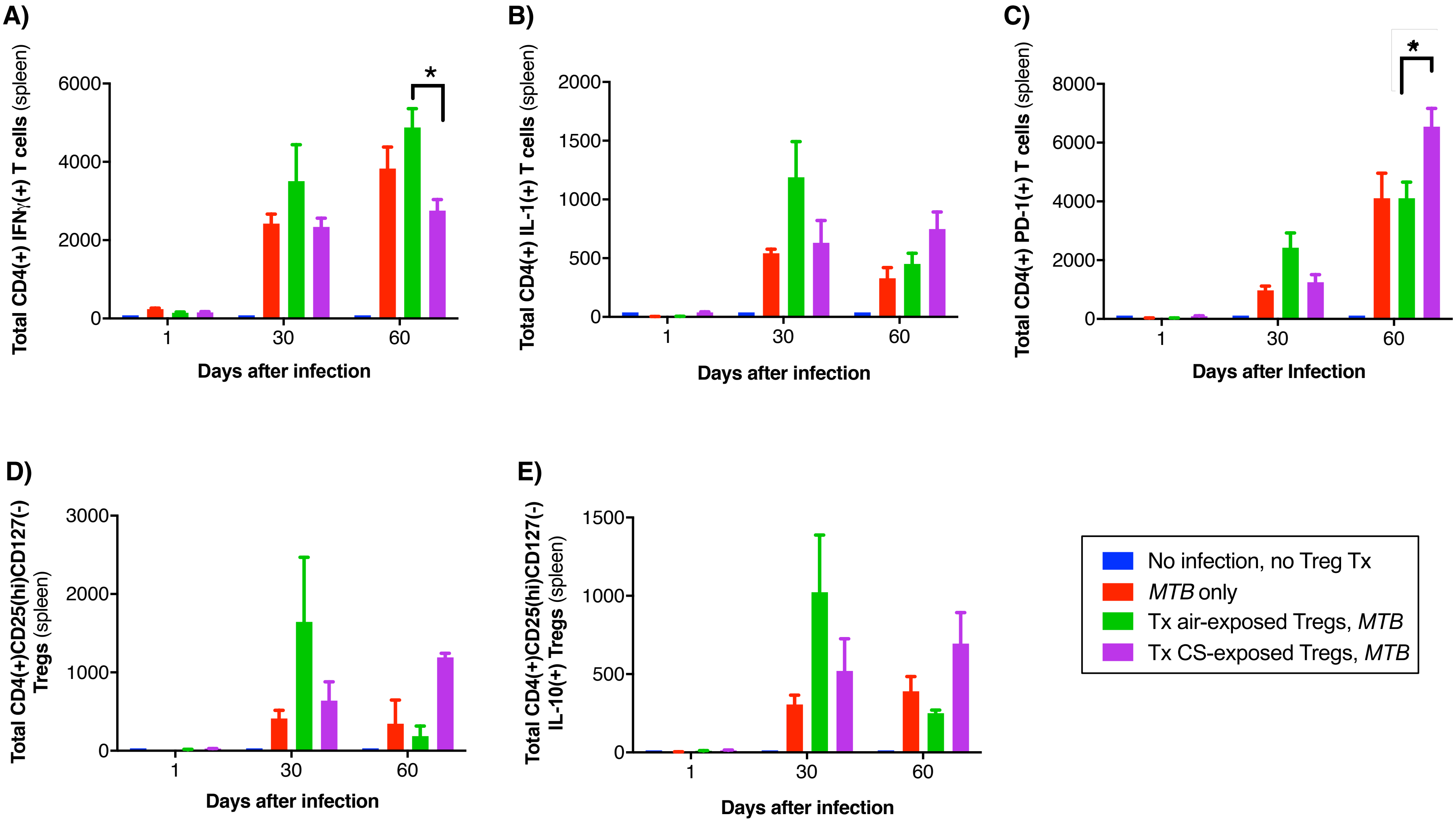

Supplemental Figure 5. Intracellular cytokine and PD-1 analyses of murine splenic T cells. Splenic $CD4^+$ T cells from uninfected mice, *MTB*-infected mice, and *MTB*-infected mice with adoptive transfer of T regulatory cells (Tregs) from either air-exposed or CS-exposed mice were stained for **(A)** IFNγ, **(B)** IL-1, and **(C)** PD-1 at the indicated time points after *MTB* infection. From the same mouse groups, **(D)** total splenic $CD4^+CD25^{hi}CD127^-$ Tregs and **(E)** $CD4^+IL\text{-}10^+$ Tregs were quantified at the indicated times. *MTB=Mycobacterium tuberculosis.*
